# Castleman Disease in an Older Patient With the Onset of Right Pleural Effusion

**DOI:** 10.7759/cureus.47035

**Published:** 2023-10-14

**Authors:** Eri Takao, Sora Matsushima, Keisuke Matsumoto, Naoto Mouri, Chiaki Sano, Ryuichi Ohta

**Affiliations:** 1 Family Medicine, Shimane University Medical School, Izumo, JPN; 2 Community Care, Unnan City Hospital, Shimane, JPN; 3 Community Medicine Management, Faculty of Medicine, Shimane University Medical School, Izumo, JPN

**Keywords:** community hospital, japan, plasma cell type, lymphadenopathy, pleural effusion, family medicine, general medicine, older patient, castleman disease

## Abstract

Castleman’s disease (CD) is an uncommon lymphoproliferative disorder with various presentations in different age groups. Although CD predominantly affects younger individuals, cases in older people are rare. The presentation of CD can range from asymptomatic to severe. We present the case of a 91-year-old male who reported dyspnea and was subsequently diagnosed with right-sided pleural effusion. The patient’s condition deteriorated despite an initial provisional diagnosis of tuberculous pleurisy and multiple interventions. A cervical lymph node biopsy later revealed a diagnosis consistent with the plasma cell type of CD. Considering the patient’s age and atypical presentation, this case adds a unique perspective to the limited literature on CD in elderly patients. Its presentation can be highly variable, and pleural effusion is rare. Our case highlights the heterogeneity of CD presentation, particularly in older age groups. The diagnosis of CD requires high suspicion, particularly in non-traditional populations. Clinicians should be aware of the varied presentations of CD, including in older patients. Unexplained pleural effusion, even in older patients, should prompt a broad differential diagnosis, including rare conditions such as CD.

## Introduction

Castleman disease (CD) is a rare, refractory lymphoproliferative disorder first reported by Benjamin Castleman in 1956 [1]. Histologically, CD is classified into two types: hyaline vascular type (HV type), characterized by lymphoid follicle proliferation, marked capillary proliferation, and hyalinization both inside and outside the follicles, and plasma cell type (PC type), characterized by clustering of numerous plasma cells between follicles [2]. The HV type accounts for approximately 90% of the cases, while the PC type accounts for approximately 10% [3]. Although the HV and PC types are mainly localized, there are reports of a multicenter (MC) type that affects multiple lymph nodes throughout the body [4]. The MC type often presents histologically similar to the PC type. The age of onset varies across all age groups, with HV and PC types commonly observed in younger individuals and the MC type predominantly observed in the older population. There was no discernible sex disparity [5]. Furthermore, based on the presence or absence of lymph node metastasis, CD is classified as unicentric CD (UCD) or multicentric CD (MCD) [1]. Although CD can manifest in a broad age range, from children to older individuals, MCD tends to be prevalent around the age of 50, and is slightly more common in males. A comprehensive survey of patients with MCD in Japan who were treated with tocilizumab between June 2005 and July 2011 revealed a median age of onset of 43 years [6]. CD is a relatively rare idiopathic lymphoproliferative disorder that frequently presents in the mediastinum or hilum of the lungs; however, it can also manifest in the cervical region [4].

In MCD, systemic symptoms such as fever, night sweats, general fatigue, weight loss, and hepatosplenomegaly are observed, together with nonspecific findings associated with chronic inflammation, such as anemia, renal dysfunction, polyclonal gammopathy, elevated C-reactive protein (CRP), and increased erythrocyte sedimentation rate (ESR) [3]. Specific clinical symptoms of MCD are rare. MCD that does not fit either category is termed idiopathic MCD, for which various treatment strategies have been considered [2]. We report a rare case of CD in an older patient with a right pleural effusion. Given its atypical progression and epidemiological rarity, this case offers information on the evaluation of pleural effusion in elderly patients.

## Case presentation

A 91-year-old male visited a rural community hospital with the chief complaint of dyspnea. The patient began to experience shortness of breath while walking three months before admission. The symptoms gradually worsened, and one week before admission, the patient needed family support in his usual life. The patient was admitted to the hospital for an assessment of dyspnea. The patient had visited the hospital a year prior, complaining of dyspnea upon exertion. During the investigation, a right pleural effusion was detected on chest computed tomography. The pleural effusion assay was categorized as transudate, and the cytology test did not show any malignancy. During one month of follow-up, the right pleural effusion disappeared naturally. The patient returned to his normal activities of daily living. His medical history included hypertension, chronic heart failure, atrial fibrillation, and ischemic heart disease. Rivaroxaban (10 mg/day) and valsartan (40 mg/day) were also administered.

The patient’s vital signs at the visit were the following: blood pressure, 145/92 mmHg; pulse rate, 100 beats/min; body temperature, 36.3°C; respiratory rate, 30 breaths/min; and oxygen saturation, 97% under 4 L of oxygen. Physical examination revealed pallor of the eyelids and conjunctivae, distention of the jugular vein in sitting position, effortful breathing, decreased breathing sounds in the right lower lung field, inspiratory crackles in the left upper lung field, and a systolic murmur at the left border of the sternum without any edema of the extremities. No other neurological abnormalities were observed. Laboratory tests revealed normocytic anemia and polyclonal gammopathy with highly inflammatory conditions of high C-reactive protein and ferritin (Table 1).

**Table 1:** Initial laboratory data of the patient CRP: C-reactive protein; Ig: immunoglobulin.

Parameter	Level	Reference
White blood cells	3.40	3.5-9.1 × 10^3^/μL
Neutrophils	74.2	44.0-72.0%
Lymphocytes	12.9	18.0-59.0%
Hemoglobin	8.7	11.3-15.2 g/dL
Hematocrit	34.2	33.4-44.9%
Mean corpuscular volume	105.8	79.0-100.0 fl
Platelets	18.8	13.0-36.9 × 10^4^/μL
Erythrocyte sedimentation rate	105	2-10 mm/hour
Total protein	8.8	6.5-8.3 g/dL
Albumin	3.2	3.8-5.3 g/dL
Total bilirubin	0.8	0.2-1.2 mg/dL
Aspartate aminotransferase	28	8-38 IU/L
Alanine aminotransferase	13	4-43 IU/L
Lactate dehydrogenase	336	121-245 U/L
Blood urea nitrogen	15.9	8-20 mg/dL
Creatinine	0.76	0.40-1.10 mg/dL
Serum Na	132	135-150 mEq/L
Serum K	3.6	3.5-5.3 mEq/L
Serum Cl	98	98-110 mEq/L
Ferritin	163.8	14.4-303.7 ng/mL
CRP	2.87	<0.30 mg/dL
IgG	3141	870-1700 mg/dL
IgM	152	35-220 mg/dL
IgA	688	110-410 mg/dL
IgE	8	<173 mg/dL
Urine test		
Leukocyte	(1+)	Negative
Protein	(1+)	Negative
Blood	(3+)	Negative

**Figure 1:**
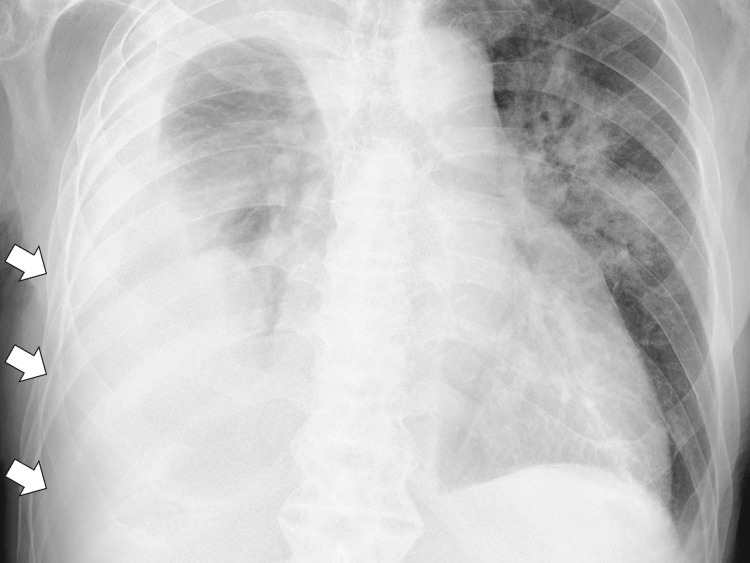
Chest X-ray showing bilateral infiltrating shadows and right pleural effusion (white arrows)

Chest radiography revealed bilateral infiltrating shadows and a right pleural effusion (Figure 1).

**Figure 2:**
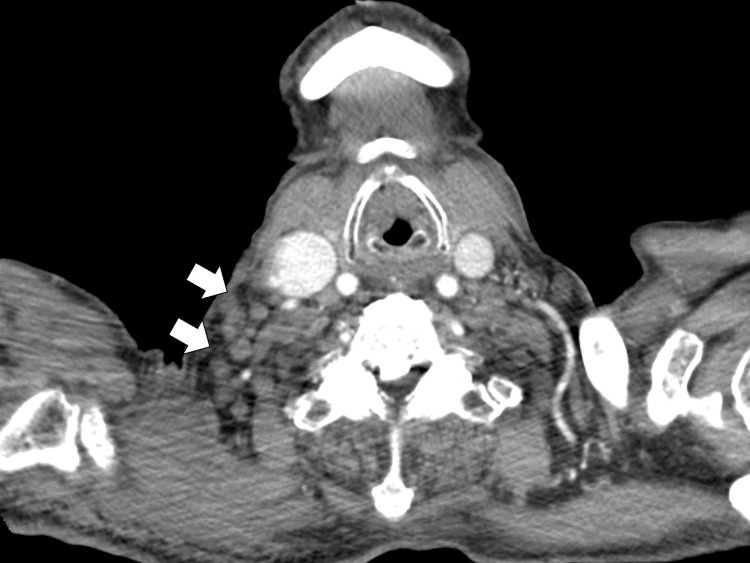
Contrast-enhanced chest computed tomography revealed multiple small lymph nodes in the right supraclavicular fossa (white arrows)

Contrast-enhanced chest computed tomography revealed multiple small lymph nodes in the right supraclavicular fossa (Figure 2).

The aspiration of the right pleural effusion yielded bloody fluid, and the results of the analysis were the following: pH 7.417, negative gram stain, erythrocyte sedimentation >50/high power field (HPF), leukocyte sedimentation 3049/HPF, total protein 3.2 g/dL, lactate dehydrogenase 1608 IU/L, and glucose 42 mg/dL, indicating an exudative pleural effusion.

The patient was diagnosed with heart failure complicated by bacterial pneumonia on the day of admission, and treatment with furosemide (20 mg/day) and ampicillin/sulbactam (12 g/day) was initiated. On the second day of admission, non-invasive positive pressure ventilation was initiated, and a right pleural drainage tube was placed. On the fourth day after admission, serum hemoglobin decreased to 7.1 g/dL, and a blood transfusion was performed. Due to expiratory wheezing on the seventh day of admission, the patient was transiently diagnosed with asthma exacerbation, and an intravenous dose of 40 mg of prednisolone was initiated. On the ninth day after admission, dobutamine was administered to treat refractory congestive heart failure. On the 10th day of admission, the patient’s respiratory condition improved after starting dobutamine administration. Further analysis of the pleural effusion revealed an increase in the adenosine deaminase level to 79 mg/dL. Therefore, a provisional diagnosis of tuberculous pleurisy was made, and anti-tubercular treatment was initiated with isoniazid, rifampin, pyrazinamide, and ethambutol. However, his respiratory condition gradually worsened on the 14th day after admission, and intubation and artificial ventilation were started. His high inflammatory condition of mild fever and high CRP level of 14 mg/dL persisted and were further investigated.

**Figure 3:**
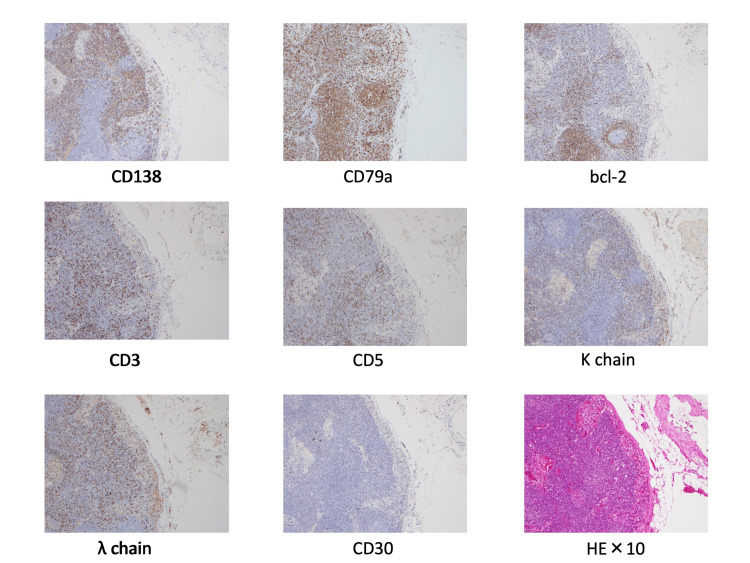
The pathology of the right cervical lymph node shows cell proliferation similar to histiocytes and lymphocytes, with a partial mixture of large cells with lymphoid follicles sporadically observed and predominantly cells positive for B-cell markers (CD 79a), with a particularly notable increase in CD138-positive plasmacytoid cells with no significant differences in the number of cells positive for κ and λ chains, cells positive for T-cell markers sporadically observed (CD 3 and 5), and Bcl-2 negative.

On the 20th day of admission, a cervical lymph node biopsy was performed to investigate progressive inflammatory conditions. The pathology of the right cervical lymph node showed the proliferation of cells resembling histiocytes and lymphocytes, with a partial mixture of large cells with sporadically observed lymphoid follicles and predominantly cells positive for B-cell markers, with a particularly notable increase in CD138-positive plasmacytoid cells and Bcl-2-negative cells. There were no significant differences in the number of cells positive for κ and λ chains; cells positive for T-cell markers were observed sporadically and Bcl-2 negative (Figure 3).

Based on the clinical course and pathological findings, the patient was diagnosed with plasma cell type CD. Tocilizumab treatment for CD was started on the 27th day after admission. His respiratory condition improved, but the patient was unable to wean off artificial ventilation, dependent on ventilators. Thus, a tracheostomy was performed. The patient was transferred to the hospital care ward for palliative care.

## Discussion

CD remains challenging to diagnose due to its rarity and nonspecific presentation. This case highlights the diagnostic conundrum associated with this disorder, particularly in unusual demographic and clinical settings.

CD is prevalent around the age of 50 years; an older patient with CD is relatively atypical, like in this case [7]. This emphasizes the importance of considering CD in the differential diagnosis of elderly patients with pleural effusion and systemic symptoms, although its prevalence in this age group is low. Our patient initially presented with dyspnea, and investigations revealed a right pleural effusion, which resolved spontaneously and then reappeared. This transient nature can mask the underlying cause and delay diagnosis. Pleural effusion, especially exudative, can be observed in many conditions, including malignancy, infections, and other inflammatory disorders [8]. In particular, the possibility of tuberculous pleurisy due to elevated levels of adenosine deaminase was considered, further complicating the diagnosis [9,10]. This highlights the importance of continually investigating refractory and recurrent cases of pleural effusion [11].

The patient’s high ESR and elevated CRP indicated a chronic inflammatory condition. Polyclonal gammopathy with elevated levels of immunoglobulin G (IgG) and IgA was consistent with the literature on MCD, supporting an eventual diagnosis [12]. However, these findings are nonspecific and can be observed in many other disorders, further highlighting diagnostic challenges [1,13]. The diagnosis of CD is based on tissue histopathology. Despite multiple clinical indicators pointing to other diagnoses, a cervical lymph node biopsy confirms the diagnosis [1]. This highlighted the need for a biopsy in unexplained chronic inflammatory conditions, particularly when the clinical picture does not fit a common diagnosis.

The response to tocilizumab, an interleukin-6 (IL-6) receptor antagonist, emphasized its role in CD treatment. IL-6 has been implicated in the pathogenesis of CD and the resulting systemic inflammatory response [14,15]. The rapid improvement in symptoms after tocilizumab administration underscores its efficacy in CD, consistent with the findings of a previous study of patients with MCD in Japan. Chronic heart failure, atrial fibrillation, and ischemic heart disease complicated the clinical presentation and management of the patient. The overlap of symptoms from comorbid conditions is essential for evaluating such complex cases.

## Conclusions

This case of a 91-year-old man diagnosed with CD underscores the challenges of identifying rare conditions, especially in elderly patients with atypical presentations. Initial diagnoses veered towards more prevalent conditions and masked the true underlying disease. The pivotal role of the cervical lymph node biopsy in this diagnosis emphasizes the importance of thorough investigation in persistent or unusual cases. This patient's journey highlights physicians' need to maintain a comprehensive differential diagnosis, especially when faced with overlapping and transient symptoms.
